# Task-related brain activity and functional connectivity in upper limb dystonia: a functional magnetic resonance imaging (fMRI) and functional near-infrared spectroscopy (fNIRS) study

**DOI:** 10.1117/1.NPh.7.4.045004

**Published:** 2020-10-19

**Authors:** Danilo Donizete de Faria, Artur José Marques Paulo, Joana Balardin, João Ricardo Sato, Edson Amaro Junior, Carlos Arruda Baltazar, Renata Prôa Dalle Lucca, Vanderci Borges, Sonia Maria Cesar Azevedo Silva, Henrique Ballalai Ferraz, Patrícia de Carvalho Aguiar

**Affiliations:** a Hospital Israelita Albert Einstein, Instituto de Ensino e Pesquisa, São Paulo, SP, Brazil; b Universidade Federal de São Paulo, Department of Neurology and Neurosurgery, R. Pedro de Toledo, São Paulo, SP, Brazil; c Hospital do Servidor Público Estadual, Vila Clementino, São Paulo, SP, Brazil; d Universidade Federal do ABC, Centro de Matemática, Computação e Cognição Av. dos Estados, Bangú, Santo André, SP, Brazil

**Keywords:** dystonia, functional near-infrared spectroscopy, functional magnetic resonance imaging, finger tapping, connectivity

## Abstract

**Significance:** Dystonia is a dynamic and complex disorder. Real-time analysis of brain activity during motor tasks may increase our knowledge on its pathophysiology. Functional near-infrared spectroscopy (fNIRS) is a noninvasive method that enables the measurement of cortical hemodynamic activity in unconstrained environments.

**Aim:** We aimed to explore the feasibility of using fNIRS for the study of task-related brain activity in dystonia. Task-related functional magnetic resonance imaging (fMRI) and resting-state functional connectivity were also analyzed.

**Approach:** Patients with idiopathic right-upper limb dystonia and controls were assessed through nonsimultaneous fMRI and fNIRS during a finger-tapping task. Seed-based connectivity analysis of resting-state fMRI was performed in both groups.

**Results:** The fMRI results suggest nonspecific activation of the cerebellum and occipital lobe in dystonia patients during the finger-tapping task with the affected hand. Moreover, fNIRS data show lower activation in terms of oxyhemoglobin and total hemoglobin in the frontal, ipsilateral cortex, and somatosensory areas during this task. In dystonia, both fMRI and fNIRS data resulted in hypoactivation of the frontal cortex during finger tapping with both hands simultaneously. Resting-state functional connectivity analysis suggests that the cerebellar somatomotor network in dystonia has an increased correlation with the medial prefrontal cortex and the paracingulate gyrus.

**Conclusions:** These data suggest that unbalanced activation of the cerebellum, somatosensory, and frontal cortical areas are associated with dystonia. To our knowledge, this is the first study using fNIRS to explore the pathophysiology of dystonia. We show that fNIRS and fMRI are complementary methods and highlight the potential of fNIRS for the study of dystonia and other movement disorders as it can overcome movement restrictions, enabling experiments in more naturalistic conditions.

## 1 Introduction

Dystonia is characterized by sustained or intermittent abnormal muscle activity resulting in twisting movements and abnormal limb postures.^1^ It can affect any region of the body, and when a single region is affected, such as hand, upper or lower limb, neck, and eyes, it is classified as focal dystonia.^1^ Regarding the etiology, dystonia can have identifiable anatomical changes and patterns of inheritance. In the absence of these conditions or other identifiable causes, dystonia is classified as idiopathic.^1^ Although the extension and phenomenology of motor symptoms are variable and may have distinct etiologies, studies suggest that most cases are characterized by unbalanced inhibitory and excitatory processing.^2^^,^^3^ While earlier studies implicated the basal nuclei in dystonia pathophysiology, there is growing evidence that dystonia is a network disorder involving the cerebellum^4^^,^^5^ and the sensorimotor cortex.^6^

The basal nuclei and the cerebellum are strongly interconnected in such a way that dysfunctions in part of this circuit might have consequences for the other.^7^ In patients with focal hand dystonia, Rothkirch et al.^8^ showed dysfunctional connectivity in cortico-basal-ganglia and cerebello-cortical network during the finger-tapping task. They also reported diminished blood-oxygen-level-dependent imaging (BOLD) response on the supplementary motor area and facilitating endogenous connectivity between the cerebellum and motor cortex. Although some findings^5^^,^^8^^,^^9^ suggest abnormal connectivity in cortico-basal ganglia and cerebello-cortical motor networks in focal dystonia, the mechanisms underlying the malfunction in these nodes are still a matter of debate.

There is increasing evidence acknowledging the involvement of the cerebellum in dystonia, suggesting that its dysfunction might underlie abnormalities of sensorimotor integration or aberrant plasticity.^5^ Cerebellar modulation of the motor cortex is altered in patients with focal dystonia^10^ and structural changes have been observed across several studies.^11^^–^^14^

The cerebellum might influence the primary cortex responsiveness by a complex mechanism with several nodes through the cerebello-thalamo-cortical pathway, and studies suggest that, through cerebellar stimulation, it might be possible to regulate the motor excitability and plasticity.^15^ Whether cerebellar abnormalities are due to compensatory changes or a cause of idiopathic dystonia demands further exploration.

Dystonia has a complex phenomenology, where subtle postural changes or sensory inputs can either trigger or improve abnormal postures. Although functional magnetic resonance imaging (fMRI) has a powerful spatial resolution, it poses challenges for the study of tasks that involve complex movements, such as walking, writing, or playing an instrument. Functional near-infrared spectroscopy (fNIRS) is a noninvasive technique that uses a hemodynamic measure based on the optical properties of oxygenated hemoglobin (oxy-Hb) and deoxygenated hemoglobin (deoxy-Hb), making indirect inferences to brain metabolism/cortical activity. Compared with fMRI, it has some advantages, such as lower cost, portability, and less sensitivity to motion artifacts, all of which could expand its use to more naturalistic experiments, bringing this kind of research to places where fMRI is not readily available. To date, fMRI is the gold standard technique for *in vivo* imaging of the brain. Still, as fNIRS presents several advantages, it can eventually become a relatively low-cost alternative for the study of motor tasks. The finger-tapping task has been shown to be highly reproducible in fMRI studies; the understanding of how fMRI compares to fNIRS in this task might show us the feasibility of using this method for the study of motor tasks. To our knowledge, fNIRS has never been used in patients with dystonia. In this work, we aimed to explore the brain’s hemodynamic response during the finger-tapping task in patients with focal upper limb dystonia, comparing its results to those of fMRI. We hypothesize that patients with dystonia have a differential activation of the cerebellum, basal nuclei, and somatomotor cortex, and altered functional connectivity in the somatomotor network. To further examine this network, we performed seed-based resting-state functional connectivity analysis based on fMRI results.

## 2 Methods

This study was approved by the Institutional Review Boards of all participating centers: Hospital Israelita Albert Einstein, Hospital Servidor Público Estadual, and Department of Neurology and Neurosurgery of Universidade Federal de São Paulo (UNIFESP). All subjects provided informed written consent.

### 2.1 Subjects

Twenty-nine patients with focal right-upper limb dystonia were recruited by neurologists at the participating movement disorders centers or by advertisement between the years 2017 and 2019. Twenty-seven healthy controls were recruited via advertisement and convenience. Inclusion criteria were age between 18 and 60 years and alphabetization in Portuguese with a minimum of 8 years of formal education. All subjects were right-handed; handedness was determined by self-report and confirmed by the Edinburgh Handedness Inventory. The diagnosis of idiopathic focal dystonia was established according to current recommendations,^1^ including a normal brain image exam [magnetic resonance or computer tomography (CT) scan] before recruitment. All dystonia patients had focal right-upper limb dystonia, 11 of which were task specific (writer’s cramp). Exclusion criteria were neurological diseases (except for the dystonia group), history of neurosurgery, moderate to severe head or upper limb trauma, cancer, uncontrolled metabolic disorders, current use of anticholinergics, neuroleptics, antidepressants, benzodiazepines, vasodilators/constrictors, and history of substance abuse. Patients under botulinum toxin treatment had to wait for at least three months after receiving the last injection to participate in the study.

Two patients with dystonia were excluded due to MRI incidental findings (microangiopathy and a brain tumor). One control was excluded due to technical problems in the functional acquisition. In total, our fMRI sample had 27 patients (18 females) and 26 controls (14 females). The mean age of dystonia patients was 44.7 years (SD = 10.94 years), and the mean age of the control group was 42.7 years [standard deviation (SD) = 11.95 years]. Eleven patients had task-specific dystonia (writer’s cramp). The median disease duration was 10 years, ranging from 1 to 35 years. Clinical and demographic characteristics are available in Tables S1 and S2 in the Supplementary Material.

### 2.2 Paradigm

The finger-tapping task consisted of 12 blocks, which contained one of three different conditions (four blocks each): right hand and left hand or both hands; each block lasted 30 s, followed by an interval of 30 s when subjects were instructed to remain still. Volunteers had to oppose the fingers to the thumb, starting from the index finger to the little finger, repeating the same sequence as fast and accurately as possible. Blocks were randomized using the E-Prime^®^ 2.0 software. The finger-tapping paradigm was the same for both fMRI and fNIRS acquisition, except for the fact that in fNIRS acquisition, participants remained seated on a chair with their hands resting on a desk. Participants were videotaped during the finger-tapping task and the investigator counted the number of finger taps to the thumb. For behavioral analysis concerning the number of finger taps for fNIRS and fMRI, we conducted a series of one-tailed independent samples t-test, with a significance level of p<0.05. Only correct finger taps were considered.

### 2.3 fMRI

#### 2.3.1 fMRI acquisition

The images were acquired on a 3.0 T equipment (Siemens^®^—PRIA) and a dedicated 64-channel reception coil. The acquisition protocol included the following sequences: quick localizer in three planes of images. Acquisition time: 35s; T1 sequence: Rapid grandient-echo (MP-RAGE), repetition time (TR): 2500 ms, echo time (TE) 3.47 ms, Inversion Time (TI): 1.100 ms, 192 slices of 1 mm of thickness without gap, and matrix of 256×256 resulting in an isotropic image of 0.5×0.5×1.0  mm. Acquisition time: 5.48 min. Functional sequence [BOLD echo planar imaging (EPI)—resting state]: TR 900 ms, TE 33 ms, flip angle 52 deg, matrix $64\times 74\;{\mathrm{mm}}^{2}$, thickness 2 mm, gap 0.6 mm, and a total of 192 volumes. Acquisition time: 6 min 11 s. Functional sequence (BOLD EPI—task of opposition of the fingers to the thumb-finger taps: TR 2000 ms, TE 25 ms, flip angle 67 deg, matrix $64\times 74\;{\mathrm{mm}}^{2}$, thickness 2.5 mm, gap 0.6 mm, and a total of 108 volumes. Acquisition time: 12 min. Resting-state images were acquired during 6 min 11 s; participants were instructed to stay awake, relaxed, with eyes open and fixed on a projected bright cross hair on a dark background.

#### 2.3.2 Task-based statistical analysis

The fMRI image processing was done by FEAT (Expert Analysis Tool), version 5.0 integrated into the FSL software [functional magnetic resonance imaging of the brain (FMRIB)’s Software Library].^16^^,^^17^ Image preprocessing sequence is as follows: movement correction using MCFLIRT, removal of noncerebral tissue using a brain extraction tool (BET),^18^ time correction of slice acquisition, special smothering using a Gaussian filter of 5 mm, normalization of general mean intensity in the four-dimensional (4-D) set data by a single multiplicative factor, high-pass filter with sigma = 50 s. The statistical analysis of the time series was performed using the FMRIB’s improved linear model tool with correction for autocorrelation. The recording of the structural high-resolution T1-weighted anatomical image and subsequent normalization for the standard Montreal Neurological Institute (MNI)152 2-mm brain were performed with the FMRIB’s linear image registration tool (FLIRT).

The statistical analysis was based on the mass univariate approach using a general linear model (GLM) regarding the response in each voxel to the experimental conditions of the task. The activity of each of the three conditions of interest (right hand, left hand, or both) was modeled as randomized activity blocks. The regressor for each condition was modeled with a box function with a duration of 30 s, considering a factorial design^19^ in which the both hands condition interacts with the remaining conditions (left hand and right hand). Subsequently, each regressor was convolved with a gamma function (time-to-peak = 6 s, standard error = 3 s). In addition to the regressors of interest, head movement parameters were also included as noninterest regressors. In each individual, to identify areas of the motor network, the contrast between each of the conditions of interest relative to the resting condition (right>resting, left>resting, and both>resting) was evaluated using a one-sample t-test, and the t values converted to Z. The group analysis was performed with a mixed-effects model using FLAME (FMRIB’s local analysis of mixed effects) stage 1.^20^^–^^22^ Differences between patients and controls were assessed by a two-tailed t-test for independent samples for each of the contrasts (right>resting, left>resting, and both hands>resting). Z statistical maps were initially thresholded using voxel clusters determined by Z>3.09, followed by a secondary threshold of 2.3, and the cluster-corrected^23^ significant threshold of p=0.05. Clusters will be described using Harvard-Oxford Cortical Structural Atlas and Cerebellar Atlas in MNI152 space after normalization with FLIRT.

#### 2.3.3 Resting-state functional connectivity processing and analysis

Data were preprocessed and analyzed using the CONN toolbox version 18.b with standard MNI152 pipeline and parameters.^24^ The pipeline consisted of realignment and unwarping, slice-timing correction, segmentation, normalization, outlier detection, and smoothing. Nuisance variables were based on scan motion censuring (discarding volumes with displacement>2  mm and global-signal z-value>9; no subjects were excluded), realignment parameters, white matter, and cerebrospinal fluid signals. Bandpass filtering (0.008 to 0.09 Hz) and nuisance variables were regressed out using a simultaneous bandpass approach.

The functional connectivity seeds were defined based on the 7-network cerebellar atlas provided by Buckner et al.^25^ derived from the fMRI data of 1000 healthy controls available at Ref. 26. The names of these cerebellar seeds resembled those of the cortical networks (i.e., somatomotor, default-mode network, visual, dorsal attention, salience, limbic, and frontoparietal). Each corresponding resting-state cerebellar network was used as a seed.

For each individual, we produced connectivity correlation maps by extracting the mean BOLD time course from voxels within each seed and computing Pearson’s correlation coefficients between that time course and the time course of all other voxels. Correlation coefficients were converted to normally distributed z-scores using the Fisher transformation to allow second-level GLM analysis. Two-sample t-tests were performed on the Fisher transformed r-maps to examine differences in resting-state functional connectivity between the dystonia and control groups. Group-level effects were considered significant if they exceeded a peak amplitude of t>3.09 and a family-wise error-corrected cluster extent threshold of p<0.05.

### 2.4 fNIRS

#### 2.4.1 fNIRS acquisition

**Fig. 1:**
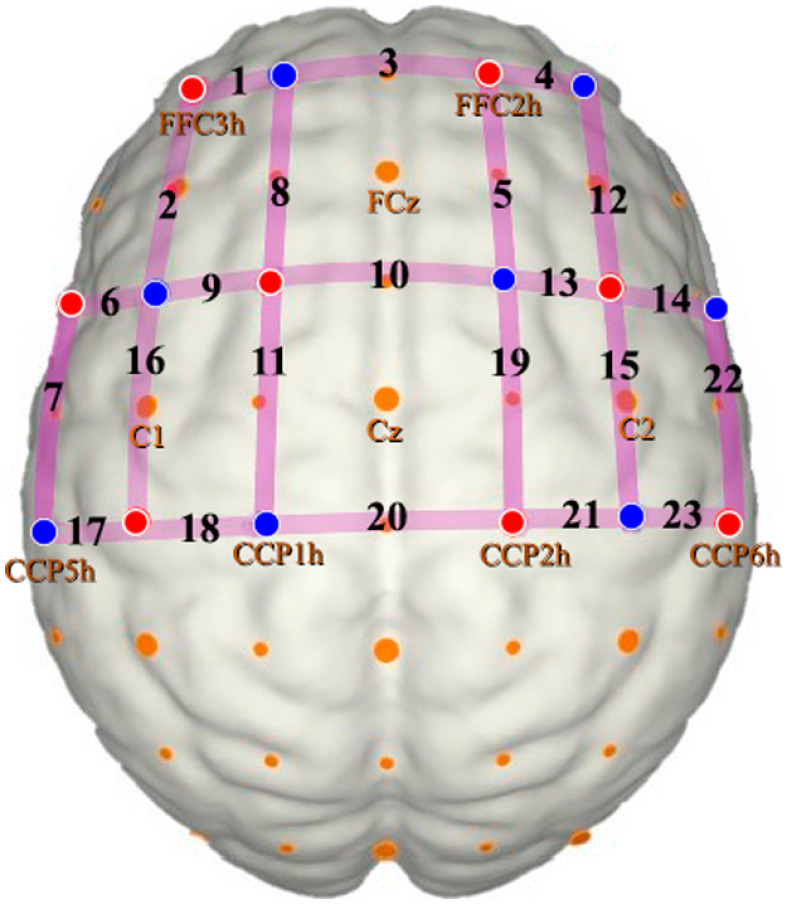
fNIRS channels set up: sources and detectors are marked in red and blue, respectively. Each channel is represented in purple lines with its respective number.

The hemodynamic signals were collected using a continuous-wave fNIRS system (NIRx Medical Technologies) with eight LED illumination sources emitting two wavelengths of near-infrared light (760 and 850 nm) and eight optical detectors. We recorded the signals at a sampling rate of 7.81 Hz. Sources and detectors were positioned bilaterally on the measuring cap with reference to the 10–5 international system.^27^ The spatial distribution of the optodes on the cap was chosen to result in channels (i.e., source–detectors pairs) with standard interoptode distances of ∼30  mm. A total of 23 channels covered regions of the middle frontal gyrus, motor, and sensorimotor cortices in addition to the supplementary motor area optodes set up are shown in Fig. 1 and corresponding anatomical landmarks are available in Table S9 in the Supplementary Material. Channels that displayed gain settings and coefficients of variation higher than 3% and 7.5%, respectively, were considered as bad channels and were excluded from the analyses (Table S10 in the Supplementary Material). Participants that had more than five bad channels were excluded. In total, 12 participants were excluded, and 21 patients and 20 controls were analyzed.

#### 2.4.2 fNIRS statistical analysis

The raw intensity data were prewhitened using the nirsLab software. The optical signals were converted to oxy-Hb and deoxy-Hb concentration changes by applying the modified Beer–Lambert equation using a differential pathlength factor of 7.25 for wavelength 1 and 6.38 for wavelength 2, molar extinction coefficients were taken from Kollias and Gratzer,^28^ which for oxy-Hb were 1486.5865 and 2526.391 and for deoxy-Hb were 3843.707 and 1798.643.

The fNIRS time series was modeled by a GLM embedded in nirsLab using a canonical hemodynamic response function (time-to-peak = 6 s, time-to-trough = 16 s). We used prewhitening autoregressive AR(n) in the intensity data, which is an autoregressive model based on iteratively reweighted least squares. This type of analysis is highly recommended to remove the effects of structured noise^29^ and to avoid false positives due to low-frequency changes in the filtered oxy-Hb and deoxy-Hb signals.

To control for false positives, p-values for statistical parametric mapping SPM{t} map were Bonferroni corrected (p>0.00217).

## 3 Results

### 3.1 Behavioral Results of the Finger-Tapping Task

Dystonia patients and controls were compared in terms of the number of taps with the right hand, left hand, and both hands. Six participants showed dystonia posture during the task (evaluated and confirmed by a neurologist). In fMRI, a significant difference was found for the right hand t(51)=1.89, p=0.032 and for both hands t(51)=1.73, p=0.045, one-tailed. Dystonia patients had a mean number of 46.7 (SD=17.9) taps per block, whereas controls had 56.2 (SD=18.9). The degree of difference in means was moderate (Cohen’s d=0.518). Accordingly, a significant difference was found for the right-hand finger-tapping in fNIRS, t(38)=2.26, p=0.015 and for both hands t(38)=3.02, p=0.004, one-tailed. The degrees of freedom are different in both analyses because 12 participants were excluded from fNIRS data due to the mentioned criteria in Sec. 2.

### 3.2 Group Comparison (fMRI)

**Fig. 2:**
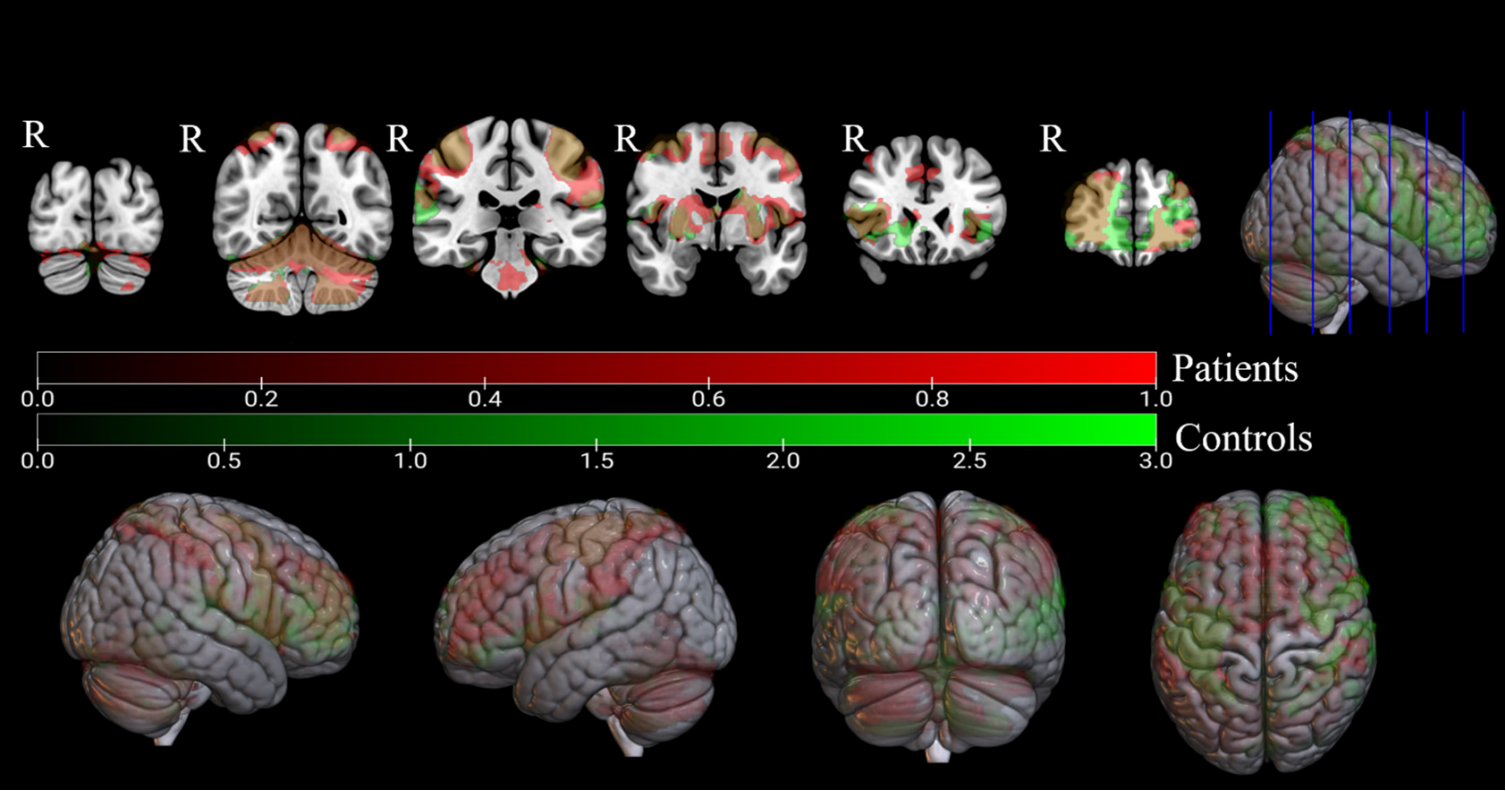
fMRI activation maps during the right-hand finger-tapping: clusters are marked in red for dystonia patients and in green for controls. Overlapping areas are shown in light brown. Coronal slices in MNI coordinates (y=−84, −56, −30, −2, 24, and 50).

Both dystonia patients and controls had bilateral cortical activation during right-hand finger tapping and, in both, the clusters peaked in the contralateral somatosensory cortex and ipsilateral cerebellum. Clusters are shown in Fig. 2 and Table 1. The contrasts regarding left-hand finger tapping are shown in Figs. S3 and S4, and Tables S5 and S6 in the Supplementary Material; and for both hands finger tapping in Figs. S5 and S6 and Tables S7 and S8 in the Supplementary Material.

**Table 1:** Activation clusters for right-hand finger tapping.

			MNI coordinates of peak voxel				
Right hand>resting	Cluster size	Z max	X	Y	Z	p	Region
Dystonia	38241	7.04	−40	−20	58	<0.001	L precentral gyrus
	14347	7.72	16	−52	−18	<0.001	R cerebellum V
	701	4.41	−52	−62	8	<0.001	L lateral occipital cortex
Controls	15523	5.44	62	14	36	<0.001	R middle frontal gyrus
	11656	6.74	−34	−22	60	<0.001	L precentral gyrus
	8123	7.16	18	50	−18	<0.001	R cerebellum V
	1404	4.13	2	0	58	<0.001	Juxtapositional lobule cortex^a^

Note: Cluster size measured in voxels. L = left; R = right.

a Formerly supplementary motor cortex.

**Fig. 3:**
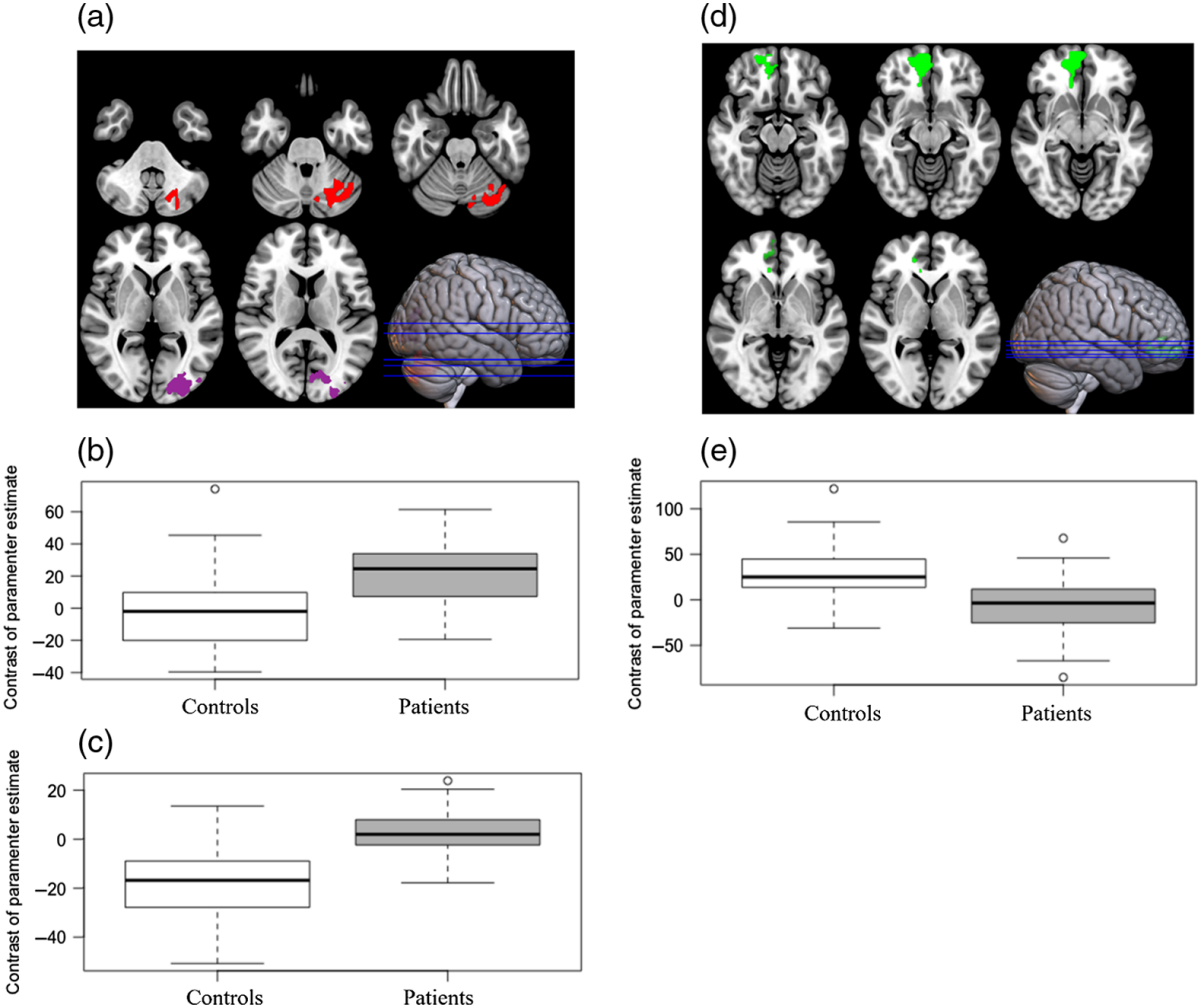
(a) Contrast dystonia > controls for right-hand finger tapping: clusters in left cerebellum (x=−24, y=−68, and z=−34; Zmax=3.54, cluster peak) and left occipital lobe (x=−28, y=−96, and z=12; Zmax=3.74, cluster peak), found in voxel-wise analyses contrasting patients and controls, axial slices in MNI coordinates (z=−40, −24, 12, and 20). (b) Boxplot of the cerebellar cluster: mean signal change relative to resting (p=0.000732). (c) Boxplot of the left occipital lobe: mean signal change relative to resting (p=0.000647). (d) Contrast controls>dystonia for both hands finger tapping: cluster in frontal pole (x=10, y=46, and z=−12) found in voxel-wise analyses (Zmax=3.57, cluster peak) contrasting controls and patients, axial slices in MNI coordinates (z=−15, −12, −8, 3, and 1). (e) Box plot of the frontal pole cluster: mean signal change relative to resting (p=0.011).

Differences between patients with upper limb dystonia and controls were assessed by voxel-wise analysis using a two-tailed independent sample t-test, a cluster-corrected significant threshold of p<0.001.^18^ No differences were detected between patients and controls using the Z>3.09 threshold in any condition. However, a threshold of Z>2.3 resulted in two clusters by contrasting dystonia>controls during right-hand finger-tapping: one in the left cerebellum (crus I, crus II, and lobule VI: segment of cerebellar posterior lobe), peak-cluster MNI coordinates −24 −68 −34 (Zmax=3.54, p=0.000732), another in the left occipital lobe (occipital pole and lateral occipital cortex, inferior and superior division), peak-cluster MNI coordinates: −28 −96 +12 (Zmax=3.74, p=0.000647), shown in Fig. 3(a); boxplots regarding the mean of contrast parameter estimate are shown in Figs. 3(b) and 3(c). For both hands condition, a cluster in the frontal cortex (frontal medial cortex and paracingulate gyrus) peak-cluster MNI coordinates +10 +46 −12, resulted in contrasting controls>patients (Zmax=3.57, p=0.011), as shown in Figs. 3(d) and 3(e). For the left-hand finger-tapping, a similar pattern was observed as for both hands condition, patients had diminished BOLD effect in the frontal cortex (frontal pole and frontal medial cortex), cluster-peak MNI coordinates +10 +62 −8 (Zmax=4.07, p<0.0001).

### 3.3 Group Comparison (fNIRS)

**Fig. 4:**
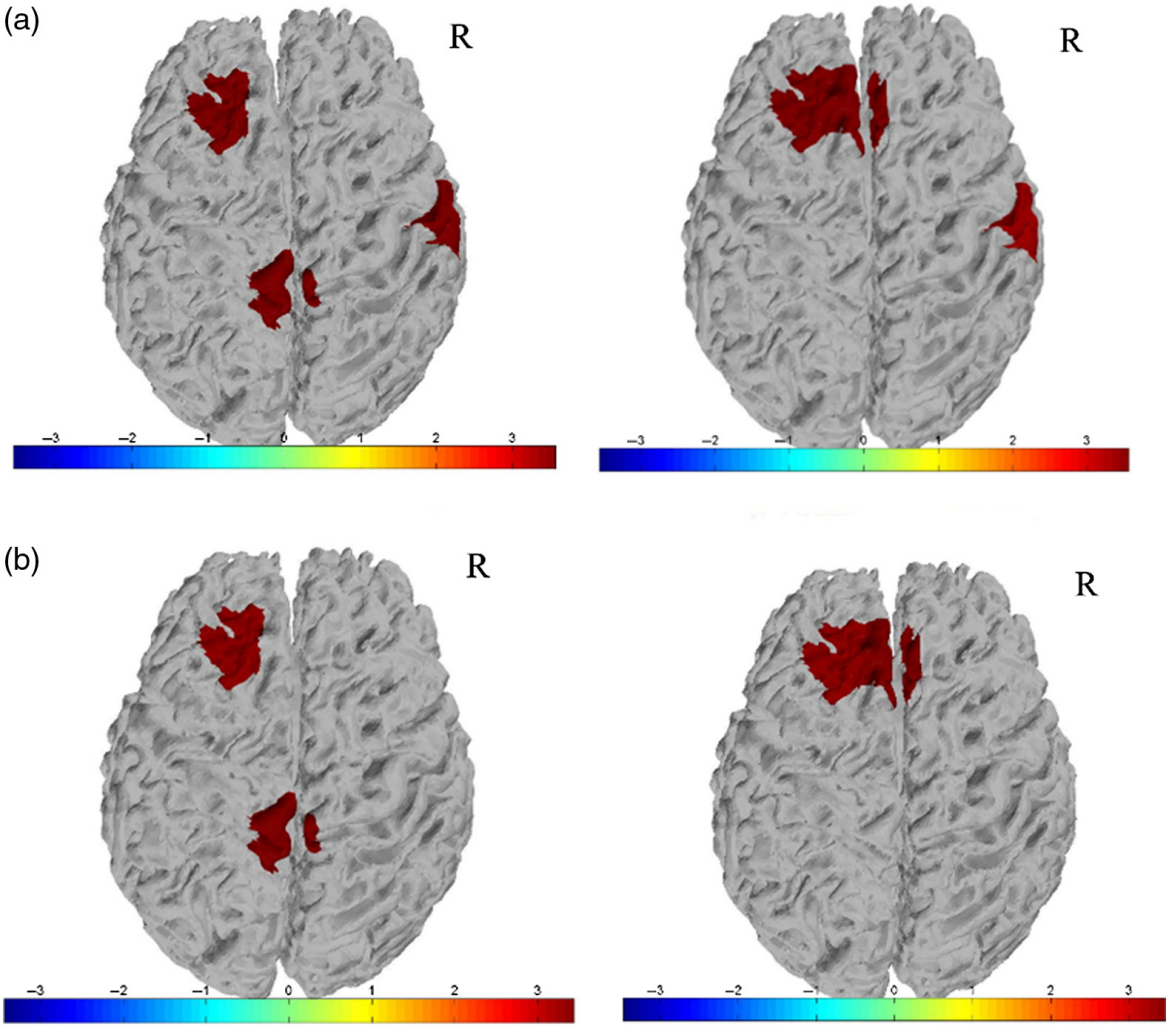
(a) Controls>patients contrast during the right-hand finger-tapping task: contrasts relative to finger tapping with the right hand for oxy-Hb (left)—left middle frontal gyrus, medial postcentral gyrus, and right supramarginal gyrus and total-Hb (right)—superior frontal gyrus and right supramarginal gyrus. (b) Controls>patients contrast during both hands finger tapping: contrasts relative to finger tapping with both hands for oxy-Hb (left)—left middle frontal gyrus, medial postcentral gyrus, and total-Hb (right)—superior frontal gyrus. Statistical maps show lower cortical activation in upper limb dystonia patients, considering Bonferroni correction ($p_{\text{thresholded}}<0.00217$). Color bars based on statistical parametric map SPM {t} image.

Patients showed a lower activation during the right hand and both hands finger-tapping tasks. For oxy-Hb contrasts, significant results comprised the left middle frontal gyrus, medial postcentral gyrus, and the right supramarginal gyrus ($p_{\text{thresholded}}<0.00217$) as shown in Fig. 4(a) (right). The contrast regarding total-Hb exhibited statistically significant differences ($p_{\text{thresholded}}<0.00217$) for the superior frontal gyrus and the right supramarginal gyrus Fig. 4(a) (left). For both hands finger tapping, a similar pattern was observed in the group comparison, but no difference was detected in the right supramarginal gyrus [Fig. 4(b)]. For the left hand finger-tapping, we did not find group differences in cortical activation for oxy-Hb and total-Hb contrasts. No difference was detected in terms of deoxy-Hb in any condition.

To ensure the results from the channel-wise analyses, we conducted a series of nonparametric two-sample t-tests (Mann–Whitney U) to compare differences in each channel and condition considering the oxy-Hb (Tables S11, S12, and S13 in the Supplementary Material), total-Hb (Tables S14, S15, and S16 in the Supplementary Material), and deoxy-Hb (Tables S17, S18, and S19 in the Supplementary Material) contrasts.

### 3.4 fMRI and fNIRS Data Description

Brain areas exhibiting cortical activation during finger tapping with the right and left hands relative to rest are shown in Fig. S7 in the Supplementary Material. In terms of BOLD effect (fMRI), oxy-Hb and deoxy-Hb contrasts (fNIRS), both patients and controls exhibited activation of areas implicated in the motor activity, including the bilateral somatosensory cortex and supplementary motor cortex. For deoxy-Hb contrasts, the finger-tapping task was associated with the contralateral somatosensory cortex in patients and controls.

### 3.5 Resting-State Functional Connectivity

The dystonia group exhibited an increased correlation between the seed of the entire cerebellar somatomotor network and a cerebellar cluster spanning the medial prefrontal cortex and paracingulate gyrus, peak-cluster MNI coordinates +14 +48 +8; cluster size: 1989; cluster p-value family-wise error: 0.001). When the other cerebellar networks were used, no other between-group differences were observed.

## 4 Discussion

Functional imaging studies of patients with dystonia provide essential information on its pathophysiology, but results are not always consistent. This could be due to heterogeneous sample recruitment, as several studies mix different types of dystonia, and to other methodological differences. In this study, we accessed the brain activity of a homogeneous sample of patients with idiopathic focal upper limb dystonia during the finger-tapping task using fMRI and fNIRS. Both methods have differences in spatial resolution. Although fNIRS has limitations to access some cortical and subcortical areas, it offers a handful of possibilities to explore dystonia and other movement disorders during tasks that cannot be studied with MRI equipment, due to movement restrictions. We chose to compare the finger-tapping task in these two methods, as this is the most commonly used paradigm to explore the motor system in fMRI studies and would give us a stronger background for comparison with fNIRS. We also explored the resting-state functional connectivity based on the results of task-fMRI, using the cerebellum as a seed.

### 4.1 Cortical and Cerebellar Activation During Task

In this study, we provide evidence of altered brain activation in dystonia patients during finger tapping with the affected limb. Dystonia patients had a lower number of finger taps with the affected limb compared with controls. However, the majority did not show a dystonic posture during the task. Some studies suggest that brain imparities in tasks that do not induce dystonia might reflect primary changes or long-standing consequences of the disorders or an inherent impairment of the motor network.^15^

Regarding functional brain differences, both fMRI and fNIRS imaging detected lower activation in the frontal cortex in dystonia patients during finger-tapping of both hands simultaneously. We found a trend of increased left occipital and cerebellar activation but decreased activation in the frontal, sensorimotor, and right parietal cortex during finger-tapping with the affected limb. The latter result is well in line with other studies that reported lower activation in the somatosensory and premotor areas in patients with focal hand dystonia during the finger-tapping task.^30^^,^^31^

Nonspecific activation in the left occipital lobe and cerebellum has been reported in patients with cervical dystonia during the finger-tapping task,^32^ suggesting unbalanced activation across a large network not limited to the somatomotor cortical network.

Cerebellar activation findings in dystonia vary across studies, possibly due to methodological differences and task-related characteristics. While some detected decreased activation with the finger-tapping task and writing,^30^^,^^33^^,^^34^ others reported increased activation for patients with cervical and myoclonus dystonia and during the writing task.^35^^–^^38^ Regarding our results, we hypothesize that the activation of the contralateral cerebellum in dystonia might reflect an additional engagement of alternative motor pathways, including the ipsilateral anterior corticospinal tract, to optimize the performance of a challenging task. This finding has been observed in healthy subjects and in parkinsonian patients under motor urgency situations, and a simple task, such as finger tapping with speed and accuracy, certainly presents an extra level of difficulty for patients with hand dystonia.^39^^,^^40^

Although the role of the cerebellum is recognized, the mechanism underlying its functions in task-dependent environments is still not fully understood. As increased or decreased activation of the cerebellum varies in literature, we believe that it is currently unattainable to determine whether functional cerebellar disparities are compensatory or disease-causing. Ideally, screening patients at risk for developing dystonia, such as asymptomatic dystonia genes mutation carriers, with a long-term follow-up, could provide some answers. When looking at neurotransmission, Pizoli et al.^41^ found that increased glutamate activity through injections of kainic acid in cerebellum induces dystonia in mice. Further, recent findings of Gallea et al.^2^ showed reduced gamma-Aminobutyric acid (GABA)-A receptors in the right cerebellum and left sensorimotor cortex in patients with focal hand dystonia, and reduced gray matter in the somatosensory cortex, which can be explained by the loss of GABA-A cells. In addition, reduced GABA levels in the sensorimotor cortex might enhance sensorimotor plasticity.^42^

### 4.2 Cerebellar Connectivity

The cerebellar cluster that was more activated in dystonia patients during task-fMRI lies mainly in posterior lobe crus I. Studies concerning the fronto-cerebellar network showed that crus I is connected to medial prefrontal cortex^26^^,^^43^ and it might be associated with executive functions.^44^

Cerebellar abnormalities in structural components, distinct activation, and alterations in functional connectivity have been associated with dystonia. Mantel et al.^45^ found reduced right cerebellar gray matter volume and diminished connectivity function with the basal nuclei in dystonia patients. In individuals with writer’s cramp, Rothkirch et al.^8^ identified stronger functional connectivity between the primary motor cortex and the ipsilateral cerebellum, but diminished putamen-cerebellum effective connectivity. Our results point to a positive correlation of the cerebellar somatomotor network with the medial prefrontal cortex and paracingulate gyrus.

### 4.3 Limitations

In this study, fMRI and fNIRS imaging were not acquired simultaneously. Subtle differences occurred during acquisition, for example, the position of the head and a different environment. Even though fNIRS optodes were placed in a measuring cap, optode positions were not digitalized for each participant, and therefore, there was no account for intersubject variability of channels’ positioning. In most cases, the finger-tapping task did not induce dystonia symptoms, which might explain why our results are not showing group differences in subcortical structures strongly related to dystonia, such as the basal nuclei.

## 5 Conclusions

Cerebellar hyperactivation during the finger-tapping task with the affected limb reinforces the role of the cerebellum in dystonia. We showed that the sensorimotor cortex and the middle frontal gyrus were hypoactive in dystonia patients during finger tapping. Patients showed increased connectivity between the cerebellum and the medial frontal lobe, which might be associated with the inherent consequences of aberrant plasticity in dystonia. This is the first study showing the feasibility of using fNIRS for the study of dystonia. Combining fMRI and fNIRS provides relevant insights for dystonia pathophysiology, which poses high spatial resolution as well as the assessment of oxy-Hb change in the cortex. Due to its portability, lower sensitivity to movement-artifacts, and cost, we emphasize the advantages of using fNIRS to explore the cortical correlates of dystonia and other movement disorders during naturalistic tasks.

## Code, Data, and Materials Availability

The data that support the findings of this study are available on request from the corresponding author.
