# Supplementary material for: Task-related brain activity and functional connectivity in upper limb dystonia: a functional magnetic resonance imaging (fMRI) and functional near-infrared spectroscopy (fNIRS) study
Source: Neurophotonics. 2020 Oct 19;7(4):045004. doi: 10.1117/1.NPh.7.4.045004 (PMC7569470; doi:10.1117/1.NPh.7.4.045004)
Supplement: Supplementary file 1 [file NPh_007_045004_SD001.docx]

**SUPPLEMENTARY MATERIAL**

**Table S1**: Clinical characteristics of patients with upper limb dystonia**.**

| Patient | Sex | Age | Disease duration (years) | Task-Specific |
| --- | --- | --- | --- | --- |
| P1 | M | 19 | 1 | No |
| P2 | F | 24 | 1 | Yes |
| P3 | F | 31 | 2 | Yes |
| P4 | F | 33 | 2 | No |
| P5 | F | 34 | 18 | No |
| P6 | F | 35 | 4 | Yes |
| P7 | F | 36 | 2 | No |
| P8 | M | 37 | 6 | No |
| P9 | M | 38 | 14 | No |
| P10 | M | 40 | 2 | Yes |
| P11 | M | 40 | 26 | No |
| P12 | M | 43 | 19 | Yes |
| P13 | F | 48 | 2 | Yes |
| P14 | F | 49 | 17 | Yes |
| P15 | F | 50 | 2 | Yes |
| P16 | M | 51 | 27 | No |
| P17 | F | 51 | 9 | Yes |
| P18 | F | 52 | 35 | Yes |
| P19 | M | 52 | 22 | Yes |
| P20 | M | 53 | 23 | No |
| P21 | F | 53 | 38 | Yes |
| P22 | F | 54 | 15 | Yes |
| P23 | F | 55 | 10 | No |
| P24 | F | 56 | 11 | No |
| P25 | F | 56 | 21 | Yes |
| P26 | F | 59 | 10 | No |
| P27 | F | 60 | 8 | No |

Note: All task-specific dystonia patients had writter’s cramp.

**Table S2:** Demographic characteristics of the control group.

| Control | Sex | Age |
| --- | --- | --- |
| C1 | F | 19 |
| C2 | M | 23 |
| C3 | M | 24 |
| C4 | F | 30 |
| C5 | M | 32 |
| C6 | M | 33 |
| C7 | F | 35 |
| C8 | F | 37 |
| C9 | M | 37 |
| C10 | F | 37 |
| C11 | M | 38 |
| C12 | F | 38 |
| C13 | F | 40 |
| C14 | F | 43 |
| C15 | M | 45 |
| C16 | M | 47 |
| C17 | F | 50 |
| C18 | F | 51 |
| C19 | F | 52 |
| C20 | M | 54 |
| C21 | F | 54 |
| C22 | F | 57 |
| C23 | M | 58 |
| C24 | M | 58 |
| C25 | F | 58 |
| C26 | M | 60 |

**
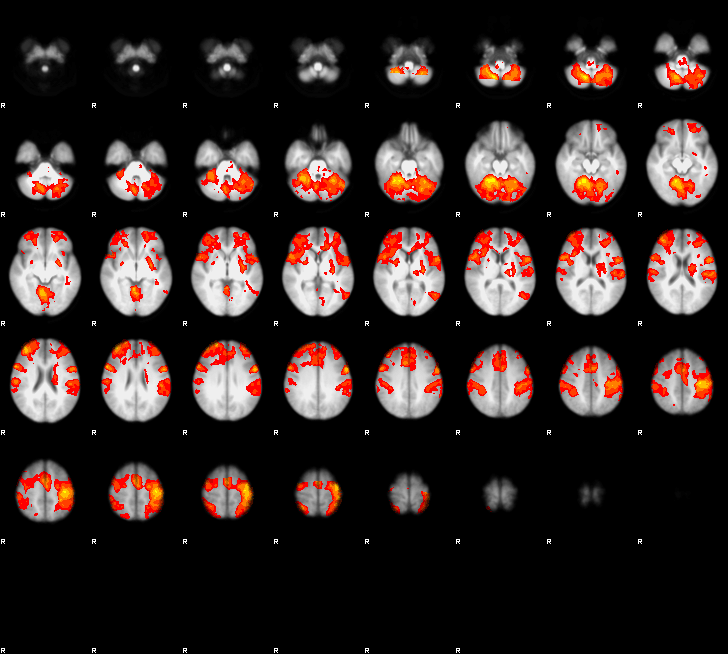
**

**Figure S1:** Activating clusters for dystonia group in right hand finger-tapping, R = right.

**Table S3:** Clusters regarding right hand finger-tapping for dystonia patients.

| **MNI Coordinates** | | | | | | |
| --- | --- | --- | --- | --- | --- | --- |
| **Cluster size** | **Z Max** | **X** | **Y** | **Z** | **P** | **Region** |
| 38241 | 7.04 | -40 | -20 | 58 | <0.001 | **L Precentral Gyrus** |
| 14347 | 7.72 | 16 | -52 | -18 | <0.001 | **R Cerebellum V** |
| 701 | 4.41 | -52 | -62 | 8 | <0.001 | **L Lateral Occipital Cortex** |

Note: MNI: Montreal Neurological Institute; Cluster measured in voxels. L = Left; R = right


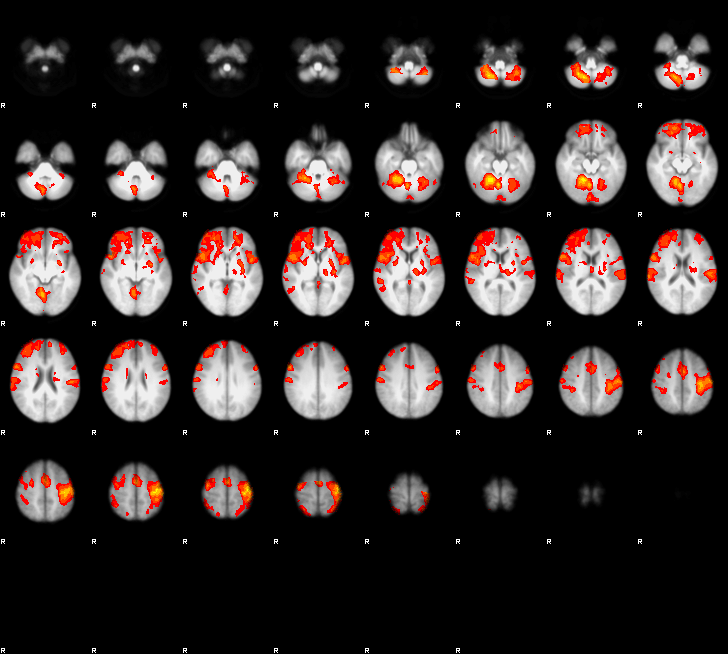


**Figure S2:** Activating clusters for control group in right hand finger-tapping, R = right

**Table S4:** Clusters regarding right hand finger-tapping for the control group.

| **MNI Coordenates** | | | | | | |
| --- | --- | --- | --- | --- | --- | --- |
| **Cluster size** | **Z Max** | **X** | **Y** | **Z** | **P** | **Region** |
| 15523 | 5.44 | 62 | 14 | 36 | <0.001 | **R Middle Frontal Gyrus** |
| 11656 | 6.74 | -34 | -22 | 60 | <0.001 | **L Precentral Gyrus** |
| 8123 | 7.16 | 18 | 50 | -18 | <0.001 | **R Cerebellum V** |
| 1404 | 4.13 | 2 | 0 | 58 | <0.001 | **Juxtapositional Lobule Cortex** |

Note: MNI: Montreal Neurological Institute; Cluster measured in voxels. L = Left; R = right


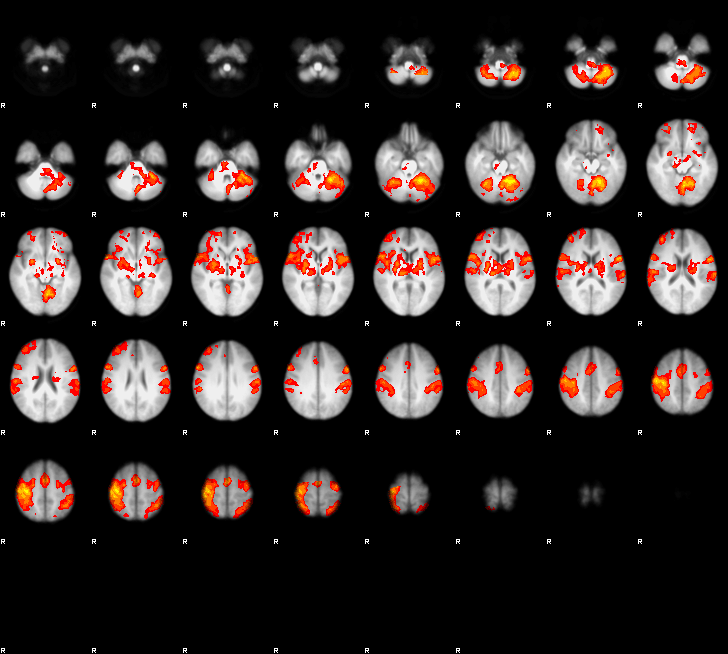


**Figure S3** Activating clusters for dystonia group in left hand finger-finger-tapping, R = right

**Tables S5:** Clusters regarding left hand finger-tapping for dystonia patients.

| **MNI Coordenates** | | | | | | |
| --- | --- | --- | --- | --- | --- | --- |
| **Cluster size** | **Z Max** | **X** | **Y** | **Z** | **P** | **Region** |
| 29681 | 7.44 | -16 | -54 | -18 | <0.001 | **L Cerebellum V** |
| 4048 | 5.12 | -66 | -22 | 18 | <0.001 | **L Postecentral Gyrus** |
| 1630 | 5.52 | -2 | 2 | 58 | <0.001 | **Juxtapositional Lobule Cortex** |
| 1206 | 32 | 32 | -62 | -20 | <0.001 | **R Cerebellum VI** |

Note: MNI: Montreal Neurological Institute; Cluster measured in voxels. L = Left; R = right


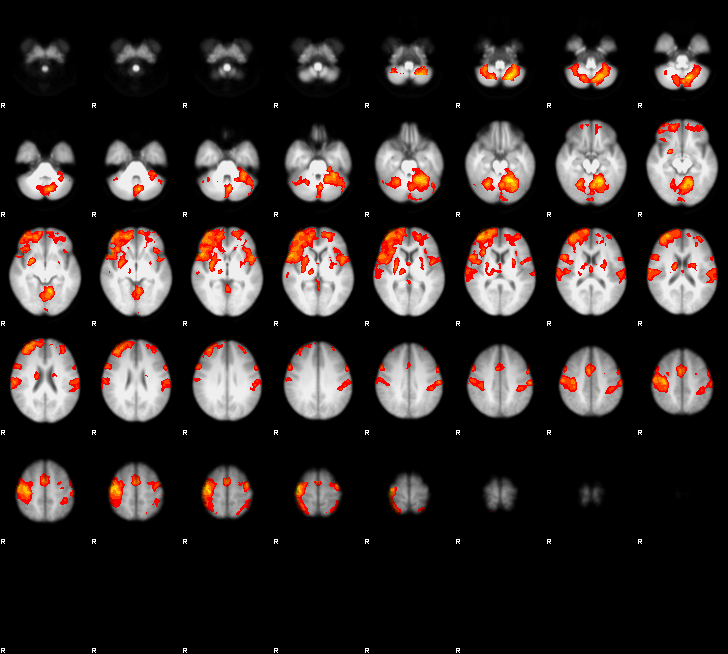


**Figure S4:** Activating clusters for control group in left hand finger-tapping, R = right.

**Table S6:** Clusters regarding left hand finger-tapping for controls.

| **MNI Coordenates** | | | | | | |
| --- | --- | --- | --- | --- | --- | --- |
| **Cluster size** | **Z Max** | **X** | **Y** | **Z** | **P** | **Region** |
| 21254 | 6.48 | 44 | -14 | 66 | <0.001 | **R Precentral Gyrus** |
| 9447 | 6.75 | -16 | -52 | -18 | <0.001 | **L Cerebellum V** |
| 5925 | 5.24 | -48 | 8 | 4 | <0.001 | **L Inferior Frontal Gyrus** |
| 1210 | 4.04 | 0 | -2 | 60 | <0.001 | **Juxtapositional Lobule Cortex** |

Note: MNI: Montreal Neurological Institute; Cluster measured in voxels. L = Left; R = right


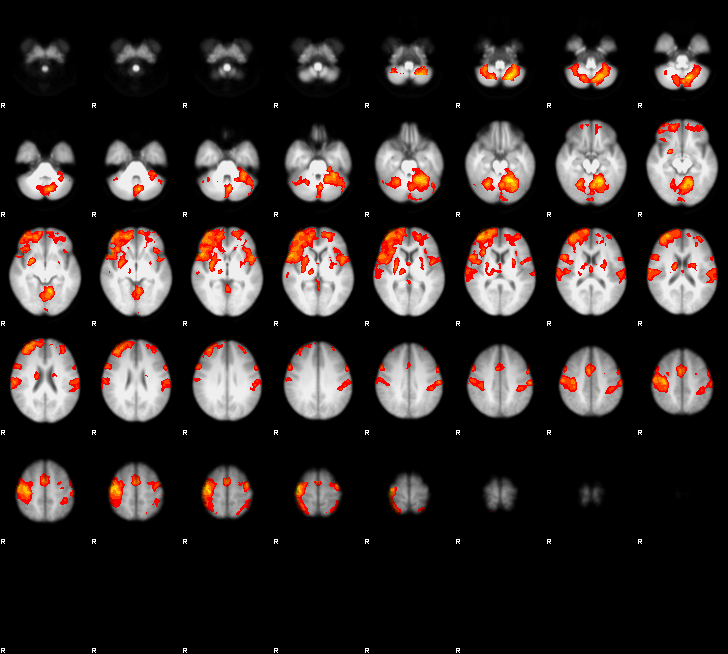


**Figure S5:** Activating clusters for dystonia group in both hands finger-tapping, R = right.

**Table S7:** Clusters regarding both hands finger-tapping for dystonia patients.

| **MNI Coordenates** | | | | | | |
| --- | --- | --- | --- | --- | --- | --- |
| **Cluster size** | **Z Max** | **X** | **Y** | **Z** | **P** | **Region** |
| 57466 | 7.06 | -18 | -56 | -18 | <0.001 | **L Cerebellum Vl** |

Note: MNI: Montreal Neurological Institute; Cluster measured in voxels. L = Left; R = right.


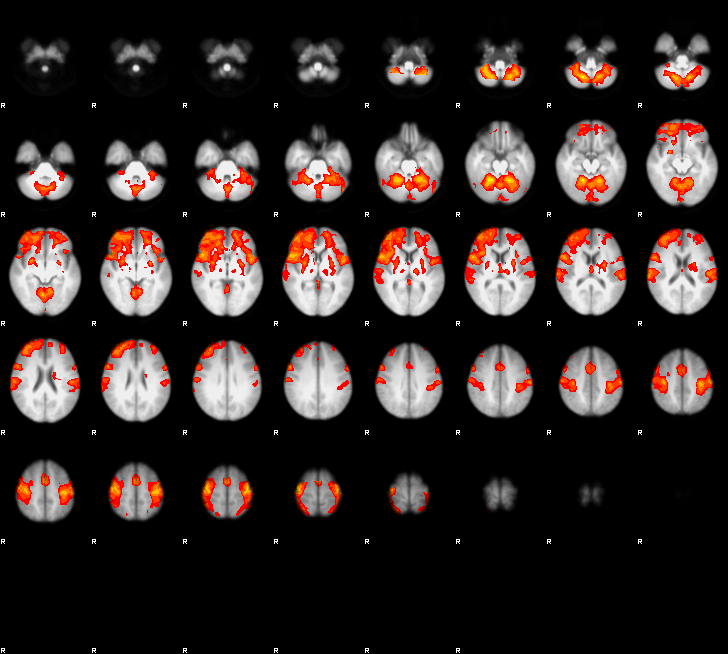


**Figure S6:** Activating clusters for control group in both hands finger-tapping

**Table S8:** Clusters regarding both hands finger-tapping for controls.

| **MNI Coordenates** | | | | | | |
| --- | --- | --- | --- | --- | --- | --- |
| **Cluster size** | **Z Max** | **X** | **Y** | **Z** | **P** | **Region** |
| 33335 | 6.20 | 42 | -14 | 66 | <0.001 | **R Inferior Temporal Gyrus** |
| 11376 | 6.64 | 18 | -50 | -18 | <0.001 | **R Cerebellum V** |
| 1260 | 3.5 | 0 | 0 | 60 | <0.001 | **Juxtapositional Lobule Cortex** |

Note: MNI: Montreal Neurological Institute; Cluster measured in voxels. L = Left; R = right

**Table S9:** Anatomical landmarks for fNIRS optodes.

| **Channel** | **Source** | **Detector** | **Channel Coordinates** | | | **Region** |
| --- | --- | --- | --- | --- | --- | --- |
|  |  |  | **X** | **Y** | **Z** |  |
| ch1 | FFC3h | FFC1h | -28 | 27 | 55 | L Middle Frontal Gyrus |
| ch2 | FFC3h | FCC3h | -38 | 12 | 55 | L Middle Frontal Gyrus |
| ch3 | FFC2h | FFC1h | 0 | 28 | 60 | Superior Frontal Gyrus |
| ch4 | FFC2h | FFC4h | 23 | 29 | 64 | R Middle Frontal Gyrus |
| ch5 | FFC2h | FCC2h | 13 | 11 | 66 | R Posterior-Medial Frontal |
| ch6 | FCC5h | FCC3h | -51 | -3 | 48 | L Middle Frontal Gyrus |
| ch7 | FCC5h | CCP5h | -60 | -19 | 38 | L Inferior Parietal Lobule |
| ch8 | FCC1h | FFC1h | -12 | 10 | 68 | L Posterior-Medial Frontal |
| ch9 | FCC1h | FCC3h | -27 | -3 | 48 | L Middle Frontal Gyrus |
| ch10 | FCC1h | FCC2h | 0 | -6 | 73 | Posterior-Medial Frontal |
| ch11 | FCC1h | CCP1h | -14 | -20 | 75 | R Posterior-Medial Frontal |
| ch12 | FCC4h | FFC4h | 37 | 12 | 55 | R Middle Frontal Gyrus |
| ch13 | FCC4h | FCC2h | 29 | -5 | 67 | R Middle Frontal Gyrus |
| ch14 | FCC4h | FCC6h | 52 | -4 | 48 | R Middle Frontal Gyrus |
| ch15 | FCC4h | CCP4h | 42 | -21 | 62 | R Inferior Parietal Lobule |
| ch16 | CCP3h | FCC3h | 42 | -20 | 61 | L Inferior Parietal Lobule |
| ch17 | CCP3h | CCP5h | -53 | -34 | 51 | L Supra Marginal Gyrus |
| ch18 | CCP3h | CCP1h | -29 | -35 | 70 | L Postcentral Gyrus |
| ch19 | CCP2h | FCC2h | 15 | -22 | 76 | R Posterior-Medial Frontal |
| ch20 | CCP2h | CCP1h | 1 | -34 | 76 | Medial Postcentral Gyrus |
| ch21 | CCP2h | CCP4h | 28 | -38 | 70 | R Postcentral Gyrus |
| ch22 | CCP6h | FCC6h | 58 | -47 | 38 | R Inferior Parietal Lobule |
| ch23 | CCP6h | CCP4h | 40 | 53 | -36 | R Supra Marginal Gyrus |

Note: Anatomical landmarks and channel coordinates (MNI Space) were extracted based on fNIRS Optodes’ Location Decider (fOLD) using Juelich Atlas

**Table S10:** Description of quality control parameters for all participants.

**Table S11:** Statistical analysis of β-contrast image (Oxy-Hb) from right hand finger-tapping.

| **Oxy-Hb (RIGHT HAND > RESTING): Dystonia Patients Vs. Controls** | | | | | | | | | | | | | |
| --- | --- | --- | --- | --- | --- | --- | --- | --- | --- | --- | --- | --- | --- |
| **Independent Sample t-test (Mann-Whitney U)** | | | | | | | | | | | | | |
|  |  |  |  |  |  |  |  |  |  |  |  |  |  |
| **Channel** | |  | | **statistic** | | **p** | | **Mean difference** | |  | | **Cohen's d** | |
| ch1 |  |  |  | 102.0 |  | 0.004 |  | 1.03e-4 |  |  |  | 0.8790 |  |
| ch2 |  |  |  | 193.0 |  | 0.670 |  | -2.28e−5 |  |  |  | -0.2091 |  |
| ch3 |  |  |  | 97.0 |  | 0.003 |  | 8.21e-5 |  |  |  | 0.9025 |  |
| ch4 |  |  |  | 104.0 |  | 0.005 |  | 1.07e-4 |  |  |  | 0.9264 |  |
| ch5 |  |  |  | 156.0 |  | 0.165 |  | 6.56e-5 |  |  |  | 0.3822 |  |
| ch6 |  |  |  | 154.0 |  | 0.149 |  | 6.75e-5 |  |  |  | 0.2759 |  |
| ch7 |  |  |  | 152.0 |  | 0.135 |  | 9.25e-5 |  |  |  | 0.5790 |  |
| ch8 |  |  |  | 161.5 |  | 0.211 |  | 4.20e-5 |  |  |  | 0.2472 |  |
| ch9 |  |  |  | 195.0 |  | 0.708 |  | 1.69e-5 |  |  |  | 0.1854 |  |
| ch10 |  |  |  | 177.0 |  | 0.401 |  | 2.20e-5 |  |  |  | 0.3407 |  |
| ch11 |  |  |  | 154.0 |  | 0.148 |  | 7.12e-5 |  |  |  | 0.4541 |  |
| ch12 |  |  |  | 189.0 |  | 0.597 |  | 2.19e-5 |  |  |  | 0.0910 |  |
| ch13 |  |  |  | 180.0 |  | 0.446 |  | 2.55e-5 |  |  |  | 0.2148 |  |
| ch14 |  |  |  | 151.5 |  | 0.130 |  | 6.09e-5 |  |  |  | 0.4715 |  |
| ch15 |  |  |  | 188.0 |  | 0.575 |  | -2.85e−5 |  |  |  | -0.2417 |  |
| ch16 |  |  |  | 183.5 |  | 0.498 |  | 7.02e-6 |  |  |  | 0.0967 |  |
| ch17 |  |  |  | 146.0 |  | 0.098 |  | 5.97e-5 |  |  |  | 0.5959 |  |
| ch18 |  |  |  | 135.0 |  | 0.052 |  | 7.73e-5 |  |  |  | 0.6054 |  |
| ch19 |  |  |  | 194.0 |  | 0.689 |  | 2.04e-5 |  |  |  | 0.1407 |  |
| ch20 |  |  |  | 82.0 |  | < .001 |  | 8.63e-5 |  |  |  | 1.0518 |  |
| ch21 |  |  |  | 164.0 |  | 0.235 |  | 4.32e-5 |  |  |  | 0.5256 |  |
| ch22 |  |  |  | 102.0 |  | 0.004 |  | 9.95e-5 |  |  |  | 0.9538 |  |
| ch23 |  |  |  | 142.0 |  | 0.078 |  | 8.06e-5 |  |  |  | 0.6978 |  |
|  | | | | | | | | | | | | | |

**Table S12:** Statistical analysis of β-contrast image (Oxy-Hb) from left hand finger-tapping.

| **Oxy-Hb (LEFT HAND > RESTING): Dystonia Patients Vs. Controls** | | | | | | | | | | | | | |
| --- | --- | --- | --- | --- | --- | --- | --- | --- | --- | --- | --- | --- | --- |
| **Independent Sample t-test (Mann-Whitney U)** | | | | | | | | | | | | | |
|  |  |  |  |  |  |  |  |  |  |  |  |  |  |
| **Channel** | |  | | **statistic** | | **p** | | **Mean difference** | |  | | **Cohen's d** | |
| ch1 |  |  |  | 161.0 |  | 0.206 |  | 4.30e-5 |  |  |  | 0.5081 |  |
| ch2 |  |  |  | 178.0 |  | 0.415 |  | 3.46e-5 |  |  |  | 0.4255 |  |
| ch3 |  |  |  | 159.0 |  | 0.190 |  | 6.31e-5 |  |  |  | 0.4093 |  |
| ch4 |  |  |  | 173.5 |  | 0.348 |  | 7.80e-5 |  |  |  | 0.3852 |  |
| ch5 |  |  |  | 138.0 |  | 0.062 |  | 1.03e-4 |  |  |  | 0.7204 |  |
| ch6 |  |  |  | 148.0 |  | 0.109 |  | 5.40e-5 |  |  |  | 0.6962 |  |
| ch7 |  |  |  | 190.0 |  | 0.615 |  | -2.26e−5 |  |  |  | -0.0103 |  |
| ch8 |  |  |  | 173.5 |  | 0.348 |  | 1.83e-5 |  |  |  | 0.3142 |  |
| ch9 |  |  |  | 99.0 |  | 0.003 |  | 9.78e-5 |  |  |  | 0.9668 |  |
| ch10 |  |  |  | 141.0 |  | 0.074 |  | 7.24e-5 |  |  |  | 0.6905 |  |
| ch11 |  |  |  | 150.0 |  | 0.122 |  | 6.78e-5 |  |  |  | 0.5774 |  |
| ch12 |  |  |  | 195.0 |  | 0.705 |  | -5.29e−5 |  |  |  | -0.0668 |  |
| ch13 |  |  |  | 137.0 |  | 0.058 |  | 7.24e-5 |  |  |  | 0.6814 |  |
| ch14 |  |  |  | 158.0 |  | 0.181 |  | 5.24e-5 |  |  |  | 0.4296 |  |
| ch15 |  |  |  | 174.0 |  | 0.354 |  | 5.37e-5 |  |  |  | 0.2669 |  |
| ch16 |  |  |  | 196.5 |  | 0.735 |  | 1.71e-5 |  |  |  | 0.0935 |  |
| ch17 |  |  |  | 195.0 |  | 0.708 |  | -1.30e−5 |  |  |  | 0.0641 |  |
| ch18 |  |  |  | 155.0 |  | 0.155 |  | 5.31e-5 |  |  |  | 0.4661 |  |
| ch19 |  |  |  | 150.0 |  | 0.122 |  | 5.14e-5 |  |  |  | 0.6109 |  |
| ch20 |  |  |  | 174.0 |  | 0.358 |  | 4.51e-5 |  |  |  | 0.4998 |  |
| ch21 |  |  |  | 203.0 |  | 0.867 |  | 5.35e-6 |  |  |  | 0.2653 |  |
| ch22 |  |  |  | 190.0 |  | 0.615 |  | 3.04e-5 |  |  |  | 0.1587 |  |
| ch23 |  |  |  | 181.0 |  | 0.461 |  | 3.64e-5 |  |  |  | 0.4605 |  |

**Table S13:** Statistical analysis of β-contrast image (Oxy-Hb) from both hands finger-tapping.

| **Oxy-Hb (BOTH HANDS > RESTING): Dystonia Patients Vs. Controls** | | | | | | | | | | | | | |
| --- | --- | --- | --- | --- | --- | --- | --- | --- | --- | --- | --- | --- | --- |
| **Independent Sample t-test (Mann-Whitney U)** | | | | | | | | | | | | | |
|  |  |  |  |  |  |  |  |  |  |  |  |  |  |
| **Channel** | |  | | **statistic** | | **p** | | **Mean difference** | |  | | **Cohen's d** | |
| ch1 |  |  |  | 101 |  | 0.004 |  | 1.46e-4 |  |  |  | 0.8760 |  |
| ch2 |  |  |  | 201 |  | 0.826 |  | -1.90e−5 |  |  |  | -0.0468 |  |
| ch3 |  |  |  | 116 |  | 0.014 |  | 1.21e-4 |  |  |  | 0.8557 |  |
| ch4 |  |  |  | 105 |  | 0.006 |  | 1.26e-4 |  |  |  | 0.8727 |  |
| ch5 |  |  |  | 124 |  | 0.025 |  | 1.28e-4 |  |  |  | 0.7456 |  |
| ch6 |  |  |  | 165 |  | 0.249 |  | 8.19e-5 |  |  |  | 0.5056 |  |
| ch7 |  |  |  | 154 |  | 0.149 |  | 8.39e-5 |  |  |  | 0.4147 |  |
| ch8 |  |  |  | 139 |  | 0.064 |  | 7.29e-5 |  |  |  | 0.6456 |  |
| ch9 |  |  |  | 165 |  | 0.249 |  | 6.53e-5 |  |  |  | 0.5891 |  |
| ch10 |  |  |  | 140 |  | 0.070 |  | 9.48e-5 |  |  |  | 0.7525 |  |
| ch11 |  |  |  | 149 |  | 0.115 |  | 9.50e-5 |  |  |  | 0.5721 |  |
| ch12 |  |  |  | 201 |  | 0.826 |  | -1.51e−5 |  |  |  | -0.0818 |  |
| ch13 |  |  |  | 155 |  | 0.157 |  | 7.17e-5 |  |  |  | 0.5453 |  |
| ch14 |  |  |  | 160 |  | 0.199 |  | 8.21e-5 |  |  |  | 0.5319 |  |
| ch15 |  |  |  | 183 |  | 0.489 |  | 4.54e-5 |  |  |  | 0.2760 |  |
| ch16 |  |  |  | 199 |  | 0.774 |  | 3.02e-5 |  |  |  | 0.1296 |  |
| ch17 |  |  |  | 140 |  | 0.070 |  | 9.27e-5 |  |  |  | 0.6169 |  |
| ch18 |  |  |  | 138 |  | 0.062 |  | 1.17e-4 |  |  |  | 0.7444 |  |
| ch19 |  |  |  | 167 |  | 0.271 |  | 7.06e-5 |  |  |  | 0.4423 |  |
| ch20 |  |  |  | 114 |  | 0.012 |  | 1.10e-4 |  |  |  | 0.8692 |  |
| ch21 |  |  |  | 163 |  | 0.228 |  | 4.92e-5 |  |  |  | 0.4802 |  |
| ch22 |  |  |  | 151 |  | 0.128 |  | 1.19e-4 |  |  |  | 0.6558 |  |
| ch23 |  |  |  | 140 |  | 0.070 |  | 1.00e-4 |  |  |  | 0.8131 |  |

**Table S14:** Statistical analysis of β-contrast image (Total-Hb) from right hand finger-tapping.

| **Total-Hb (RIGHT HAND > RESTING): Dystonia Patients Vs. Controls** | | | | | | | | | | | | | |
| --- | --- | --- | --- | --- | --- | --- | --- | --- | --- | --- | --- | --- | --- |
| **Independent Sample t-test (Mann-Whitney U)** | | | | | | | | | | | | | |
|  |  |  |  |  |  |  |  |  |  |  |  |  |  |
| **Channel** | |  | | **statistic** | | **p** | | **Mean difference** | |  | | **Cohen's d** | |
| ch1 |  |  |  | 76.0 |  | < .001 |  | 1.22e-4 |  |  |  | 1.1112 |  |
| ch2 |  |  |  | 196.0 |  | 0.728 |  | 1.84e-5 |  |  |  | 0.0418 |  |
| ch3 |  |  |  | 103.0 |  | 0.005 |  | 9.69e-5 |  |  |  | 1.0649 |  |
| ch4 |  |  |  | 104.0 |  | 0.005 |  | 1.12e-4 |  |  |  | 0.9703 |  |
| ch5 |  |  |  | 150.0 |  | 0.122 |  | 6.61e-5 |  |  |  | 0.4293 |  |
| ch6 |  |  |  | 133.0 |  | 0.045 |  | 8.99e-5 |  |  |  | 0.6091 |  |
| ch7 |  |  |  | 125.0 |  | 0.026 |  | 1.26e-4 |  |  |  | 0.8325 |  |
| ch8 |  |  |  | 141.5 |  | 0.076 |  | 6.82e-5 |  |  |  | 0.4872 |  |
| ch9 |  |  |  | 179.0 |  | 0.430 |  | 3.19e-5 |  |  |  | 0.4010 |  |
| ch10 |  |  |  | 132.0 |  | 0.042 |  | 5.39e-5 |  |  |  | 0.7473 |  |
| ch11 |  |  |  | 142.0 |  | 0.078 |  | 2.67e-5 |  |  |  | 0.4955 |  |
| ch12 |  |  |  | 162.0 |  | 0.218 |  | 3.66e-5 |  |  |  | 0.3546 |  |
| ch13 |  |  |  | 162.0 |  | 0.218 |  | 4.62e-5 |  |  |  | 0.4919 |  |
| ch14 |  |  |  | 132.0 |  | 0.042 |  | 6.93e-5 |  |  |  | 0.7522 |  |
| ch15 |  |  |  | 176.5 |  | 0.389 |  | 3.52e-5 |  |  |  | 0.2300 |  |
| ch16 |  |  |  | 169.5 |  | 0.297 |  | 4.54e-5 |  |  |  | 0.4038 |  |
| ch17 |  |  |  | 104.0 |  | 0.006 |  | 1.07e-4 |  |  |  | 0.9872 |  |
| ch18 |  |  |  | 119.0 |  | 0.018 |  | 7.28e-5 |  |  |  | 0.8015 |  |
| ch19 |  |  |  | 166.0 |  | 0.260 |  | 3.56e-5 |  |  |  | 0.3432 |  |
| ch20 |  |  |  | 86.0 |  | < .001 |  | 7.69e-5 |  |  |  | 1.0115 |  |
| ch21 |  |  |  | 135.0 |  | 0.051 |  | 4.39e-5 |  |  |  | 0.6295 |  |
| ch22 |  |  |  | 100.0 |  | 0.004 |  | 1.09e-4 |  |  |  | 1.0476 |  |
| ch23 |  |  |  | 123.0 |  | 0.023 |  | 9.96e-5 |  |  |  | 0.8032 |  |
|  | | | | | | | | | | | | | |

**Table S15**: Statistical analysis of β-contrast image (Total-Hb) from left hand finger-tapping**.**

| **Total-Hb (LEFT HAND > RESTING): Dystonia Patients Vs. Controls** | | | | | | | | | | | | | |
| --- | --- | --- | --- | --- | --- | --- | --- | --- | --- | --- | --- | --- | --- |
| **Independent Sample t-test (Mann-Whitney U)** | | | | | | | | | | | | | |
|  |  |  |  |  |  |  |  |  |  |  |  |  |  |
| **Channel** | |  | | **statistic** | | **p** | | **Mean difference** | |  | | **Cohen's d** | |
| ch1 |  |  |  | 169 |  | 0.294 |  | 3.26e-5 |  |  |  | 0.33608 |  |
| ch2 |  |  |  | 204 |  | 0.887 |  | -5.80e−6 |  |  |  | -0.10134 |  |
| ch3 |  |  |  | 144 |  | 0.088 |  | 6.21e-5 |  |  |  | 0.34375 |  |
| ch4 |  |  |  | 173 |  | 0.345 |  | 4.28e-5 |  |  |  | 0.23582 |  |
| ch5 |  |  |  | 138 |  | 0.062 |  | 1.17e-4 |  |  |  | 0.57942 |  |
| ch6 |  |  |  | 201 |  | 0.826 |  | 1.21e-5 |  |  |  | 0.14085 |  |
| ch7 |  |  |  | 184 |  | 0.510 |  | -2.54e−5 |  |  |  | -0.44208 |  |
| ch8 |  |  |  | 152 |  | 0.130 |  | 5.43e-5 |  |  |  | 0.35171 |  |
| ch9 |  |  |  | 157 |  | 0.173 |  | 5.17e-5 |  |  |  | 0.49323 |  |
| ch10 |  |  |  | 167 |  | 0.271 |  | 4.74e-5 |  |  |  | 0.46013 |  |
| ch11 |  |  |  | 165 |  | 0.249 |  | 5.74e-5 |  |  |  | 0.44606 |  |
| ch12 |  |  |  | 174 |  | 0.358 |  | -5.22e−5 |  |  |  | -0.39336 |  |
| ch13 |  |  |  | 168 |  | 0.282 |  | 4.82e-5 |  |  |  | 0.32977 |  |
| ch14 |  |  |  | 183 |  | 0.494 |  | 2.99e-5 |  |  |  | 0.18670 |  |
| ch15 |  |  |  | 188 |  | 0.575 |  | 3.09e-5 |  |  |  | 0.14971 |  |
| ch16 |  |  |  | 178 |  | 0.404 |  | -3.25e−5 |  |  |  | -0.21942 |  |
| ch17 |  |  |  | 207 |  | 0.949 |  | -2.29e−6 |  |  |  | 0.00197 |  |
| ch18 |  |  |  | 169 |  | 0.291 |  | 3.58e-5 |  |  |  | 0.41969 |  |
| ch19 |  |  |  | 182 |  | 0.465 |  | 3.63e-5 |  |  |  | 0.32801 |  |
| ch20 |  |  |  | 171 |  | 0.319 |  | 3.35e-5 |  |  |  | 0.39515 |  |
| ch21 |  |  |  | 209 |  | 0.990 |  | -5.50e−7 |  |  |  | 0.17397 |  |
| ch22 |  |  |  | 198 |  | 0.767 |  | 1.46e-5 |  |  |  | 0.02460 |  |
| ch23 |  |  |  | 175 |  | 0.372 |  | 3.42e-5 |  |  |  | 0.38066 |  |
|  | | | | | | | | | | | | | |

**Table S16:** Statistical analysis of β-contrast image (Total-Hb) from both hands finger-tapping.

| **Total-Hb (BOTH HANDS > RESTING): Dystonia Patients Vs. Controls** | | | | | | | | | | | | | |
| --- | --- | --- | --- | --- | --- | --- | --- | --- | --- | --- | --- | --- | --- |
| **Independent Sample t-test (Mann-Whitney U)** | | | | | | | | | | | | | |
| **Channel** | |  | | **statistic** | | **p** | | **Mean difference** | |  | | **Cohen's d** | |
| ch1 |  |  |  | 101 |  | 0.004 |  | 1.46e-4 |  |  |  | 0.8760 |  |
| ch2 |  |  |  | 201 |  | 0.826 |  | -1.90e−5 |  |  |  | -0.0468 |  |
| ch3 |  |  |  | 116 |  | 0.014 |  | 1.21e-4 |  |  |  | 0.8557 |  |
| ch4 |  |  |  | 105 |  | 0.006 |  | 1.26e-4 |  |  |  | 0.8727 |  |
| ch5 |  |  |  | 124 |  | 0.025 |  | 1.28e-4 |  |  |  | 0.7456 |  |
| ch6 |  |  |  | 165 |  | 0.249 |  | 8.19e-5 |  |  |  | 0.5056 |  |
| ch7 |  |  |  | 154 |  | 0.149 |  | 8.39e-5 |  |  |  | 0.4147 |  |
| ch8 |  |  |  | 139 |  | 0.064 |  | 7.29e-5 |  |  |  | 0.6456 |  |
| ch9 |  |  |  | 165 |  | 0.249 |  | 6.53e-5 |  |  |  | 0.5891 |  |
| ch10 |  |  |  | 140 |  | 0.070 |  | 9.48e-5 |  |  |  | 0.7525 |  |
| ch11 |  |  |  | 149 |  | 0.115 |  | 9.50e-5 |  |  |  | 0.5721 |  |
| ch12 |  |  |  | 201 |  | 0.826 |  | -1.51e−5 |  |  |  | -0.0818 |  |
| ch13 |  |  |  | 155 |  | 0.157 |  | 7.17e-5 |  |  |  | 0.5453 |  |
| ch14 |  |  |  | 160 |  | 0.199 |  | 8.21e-5 |  |  |  | 0.5319 |  |
| ch15 |  |  |  | 183 |  | 0.489 |  | 4.54e-5 |  |  |  | 0.2760 |  |
| ch16 |  |  |  | 199 |  | 0.774 |  | 3.02e-5 |  |  |  | 0.1296 |  |
| ch17 |  |  |  | 140 |  | 0.070 |  | 9.27e-5 |  |  |  | 0.6169 |  |
| ch18 |  |  |  | 138 |  | 0.062 |  | 1.17e-4 |  |  |  | 0.7444 |  |
| ch19 |  |  |  | 167 |  | 0.271 |  | 7.06e-5 |  |  |  | 0.4423 |  |
| ch20 |  |  |  | 114 |  | 0.012 |  | 1.10e-4 |  |  |  | 0.8692 |  |
| ch21 |  |  |  | 163 |  | 0.228 |  | 4.92e-5 |  |  |  | 0.4802 |  |
| ch22 |  |  |  | 151 |  | 0.128 |  | 1.19e-4 |  |  |  | 0.6558 |  |
| ch23 |  |  |  | 140 |  | 0.070 |  | 1.00e-4 |  |  |  | 0.8131 |  |
|  | | | | | | | | | | | | | |

**Table S17**: Statistical analysis of β-contrast image (Deoxy-Hb) from right hand finger-tapping.

| **Deoxy-Hb (RIGHT HAND > RESTING): Dystonia Patients Vs. Controls** | | | | | | | | | | | | | |
| --- | --- | --- | --- | --- | --- | --- | --- | --- | --- | --- | --- | --- | --- |
| **Independent Sample t-test (Mann-Whitney U)** | | | | | | | | | | | | | |
|  |  |  |  |  |  |  |  |  |  |  |  |  |  |
| **Channel** | |  | | **statistic** | | **p** | | **Mean difference** | |  | | **Cohen's d** | |
| ch1 |  |  |  | 181 |  | 0.461 |  | -6.23e−6 |  |  |  | -0.25883 |  |
| ch2 |  |  |  | 194 |  | 0.689 |  | -5.46e−6 |  |  |  | 0.04126 |  |
| ch3 |  |  |  | 183 |  | 0.494 |  | -5.38e−6 |  |  |  | -0.06661 |  |
| ch4 |  |  |  | 194 |  | 0.686 |  | -7.49e−6 |  |  |  | -0.13791 |  |
| ch5 |  |  |  | 153 |  | 0.142 |  | -2.28e−5 |  |  |  | -0.49692 |  |
| ch6 |  |  |  | 198 |  | 0.767 |  | -3.45e−6 |  |  |  | -0.08817 |  |
| ch7 |  |  |  | 177 |  | 0.401 |  | -1.03e−5 |  |  |  | -0.35248 |  |
| ch8 |  |  |  | 176 |  | 0.375 |  | -3.34e−5 |  |  |  | -0.23631 |  |
| ch9 |  |  |  | 188 |  | 0.566 |  | -2.63e−5 |  |  |  | 0.03760 |  |
| ch10 |  |  |  | 137 |  | 0.059 |  | -2.53e−5 |  |  |  | -0.61342 |  |
| ch11 |  |  |  | 187 |  | 0.561 |  | -7.23e−6 |  |  |  | -0.12368 |  |
| ch12 |  |  |  | 206 |  | 0.928 |  | -1.75e−6 |  |  |  | -0.17260 |  |
| ch13 |  |  |  | 170 |  | 0.297 |  | 1.13e-5 |  |  |  | -0.24121 |  |
| ch14 |  |  |  | 181 |  | 0.457 |  | 8.27e-6 |  |  |  | -0.09550 |  |
| ch15 |  |  |  | 162 |  | 0.215 |  | -2.71e−5 |  |  |  | -0.58716 |  |
| ch16 |  |  |  | 209 |  | 0.979 |  | 1.22e-5 |  |  |  | -0.00270 |  |
| ch17 |  |  |  | 184 |  | 0.510 |  | -8.51e−6 |  |  |  | -0.07680 |  |
| ch18 |  |  |  | 209 |  | 0.990 |  | 1.85e-5 |  |  |  | 0.12819 |  |
| ch19 |  |  |  | 174 |  | 0.358 |  | -7.87e−6 |  |  |  | -0.24187 |  |
| ch20 |  |  |  | 125 |  | 0.026 |  | -1.14e−5 |  |  |  | -0.66031 |  |
| ch21 |  |  |  | 179 |  | 0.430 |  | -5.90e−6 |  |  |  | -0.19761 |  |
| ch22 |  |  |  | 182 |  | 0.477 |  | 7.91e-6 |  |  |  | 0.08263 |  |
| ch23 |  |  |  | 172 |  | 0.328 |  | 8.17e-6 |  |  |  | 0.31302 |  |
|  | | | | | | | | | | | | | |

**Table S18:** Statistical analysis of β-contrast image (Deoxy-Hb) from left hand finger-tapping.

| **Deoxy-Hb (LEFT HAND > RESTING): Dystonia Patients Vs. Controls** | | | | | | | | | | | | | |
| --- | --- | --- | --- | --- | --- | --- | --- | --- | --- | --- | --- | --- | --- |
| **Independent Sample t-test (Mann-Whitney U)** | | | | | | | | | | | | | |
|  |  |  |  |  |  |  |  |  |  |  |  |  |  |
| **Channel** | |  | | **statistic** | | **p** | | **Mean difference** | |  | | **Cohen's d** | |
| ch1 |  |  |  | 200 |  | 0.806 |  | 1.23e-6 |  |  |  | 0.0931 |  |
| ch2 |  |  |  | 193 |  | 0.657 |  | 2.15e-5 |  |  |  | 0.2180 |  |
| ch3 |  |  |  | 151 |  | 0.124 |  | 2.06e-5 |  |  |  | 0.5396 |  |
| ch4 |  |  |  | 202 |  | 0.847 |  | 1.59e-6 |  |  |  | 0.1613 |  |
| ch5 |  |  |  | 121 |  | 0.020 |  | 3.76e-5 |  |  |  | 0.6802 |  |
| ch6 |  |  |  | 147 |  | 0.100 |  | 1.76e-5 |  |  |  | 0.5734 |  |
| ch7 |  |  |  | 179 |  | 0.419 |  | 3.33e-6 |  |  |  | 0.1875 |  |
| ch8 |  |  |  | 193 |  | 0.667 |  | 1.13e-5 |  |  |  | -0.0649 |  |
| ch9 |  |  |  | 135 |  | 0.050 |  | 5.81e-5 |  |  |  | 0.5607 |  |
| ch10 |  |  |  | 166 |  | 0.251 |  | 3.53e-5 |  |  |  | 0.1259 |  |
| ch11 |  |  |  | 159 |  | 0.190 |  | 1.25e-5 |  |  |  | 0.1597 |  |
| ch12 |  |  |  | 202 |  | 0.847 |  | 4.35e-6 |  |  |  | 0.1549 |  |
| ch13 |  |  |  | 181 |  | 0.461 |  | 1.07e-5 |  |  |  | 0.3615 |  |
| ch14 |  |  |  | 158 |  | 0.181 |  | 2.60e-5 |  |  |  | 0.5398 |  |
| ch15 |  |  |  | 173 |  | 0.341 |  | -1.91e−6 |  |  |  | 0.1928 |  |
| ch16 |  |  |  | 189 |  | 0.593 |  | 2.32e-5 |  |  |  | -0.0535 |  |
| ch17 |  |  |  | 208 |  | 0.969 |  | 1.09e-5 |  |  |  | -0.1300 |  |
| ch18 |  |  |  | 158 |  | 0.179 |  | 1.65e-5 |  |  |  | 0.4054 |  |
| ch19 |  |  |  | 193 |  | 0.670 |  | 5.25e-6 |  |  |  | 0.1446 |  |
| ch20 |  |  |  | 201 |  | 0.826 |  | 1.40e-6 |  |  |  | 0.0590 |  |
| ch21 |  |  |  | 203 |  | 0.855 |  | -1.18e−5 |  |  |  | -0.0407 |  |
| ch22 |  |  |  | 210 |  | 1.000 |  | -5.00e−8 |  |  |  | 0.1308 |  |
| ch23 |  |  |  | 188 |  | 0.579 |  | 5.78e-6 |  |  |  | 0.2979 |  |
|  | | | | | | | | | | | | | |

**Table S19:** Statistical analysis of β-contrast image (Deoxy-Hb) from both hands finger-tapping.

| **Deoxy-Hb (BOTH HANDS > RESTING): Dystonia Patients Vs. Controls** | | | | | | | | | | | | | |
| --- | --- | --- | --- | --- | --- | --- | --- | --- | --- | --- | --- | --- | --- |
| **Independent Sample t-test (Mann-Whitney U)** | | | | | | | | | | | | | |
|  |  |  |  |  |  |  |  |  |  |  |  |  |  |
| **Channel** | |  | | **statistic** | | **p** | | **Mean difference** | |  | | **Cohen's d** | |
| ch1 |  |  |  | 186 |  | 0.544 |  | -6.11e−6 |  |  |  | -0.11764 |  |
| ch2 |  |  |  | 206 |  | 0.928 |  | 7.95e-7 |  |  |  | 0.14036 |  |
| ch3 |  |  |  | 195 |  | 0.708 |  | 5.10e-6 |  |  |  | 0.24639 |  |
| ch4 |  |  |  | 197 |  | 0.735 |  | -3.24e−6 |  |  |  | 0.03592 |  |
| ch5 |  |  |  | 198 |  | 0.767 |  | 6.63e-6 |  |  |  | 0.16485 |  |
| ch6 |  |  |  | 180 |  | 0.446 |  | 1.91e-5 |  |  |  | 0.16541 |  |
| ch7 |  |  |  | 208 |  | 0.958 |  | 2.12e-5 |  |  |  | -0.11613 |  |
| ch8 |  |  |  | 207 |  | 0.938 |  | 1.84e-5 |  |  |  | -0.18516 |  |
| ch9 |  |  |  | 188 |  | 0.579 |  | 1.37e-5 |  |  |  | 0.30271 |  |
| ch10 |  |  |  | 196 |  | 0.715 |  | -1.92e−5 |  |  |  | -0.20442 |  |
| ch11 |  |  |  | 185 |  | 0.514 |  | 1.23e-5 |  |  |  | 0.03178 |  |
| ch12 |  |  |  | 197 |  | 0.747 |  | 4.84e-6 |  |  |  | 0.00996 |  |
| ch13 |  |  |  | 206 |  | 0.917 |  | -2.08e−5 |  |  |  | 0.12170 |  |
| ch14 |  |  |  | 171 |  | 0.315 |  | 1.22e-5 |  |  |  | 0.33582 |  |
| ch15 |  |  |  | 201 |  | 0.825 |  | -1.09e−5 |  |  |  | -0.11124 |  |
| ch16 |  |  |  | 209 |  | 0.979 |  | 2.28e-5 |  |  |  | -0.02966 |  |
| ch17 |  |  |  | 195 |  | 0.696 |  | 2.56e-5 |  |  |  | -0.12091 |  |
| ch18 |  |  |  | 185 |  | 0.523 |  | 4.12e-5 |  |  |  | 0.31737 |  |
| ch19 |  |  |  | 208 |  | 0.969 |  | -1.55e−6 |  |  |  | -0.00590 |  |
| ch20 |  |  |  | 171 |  | 0.309 |  | -3.53e−5 |  |  |  | -0.38531 |  |
| ch21 |  |  |  | 196 |  | 0.728 |  | -5.36e−6 |  |  |  | -0.15077 |  |
| ch22 |  |  |  | 204 |  | 0.887 |  | 5.85e-6 |  |  |  | 0.12473 |  |
| ch23 |  |  |  | 186 |  | 0.544 |  | 1.05e-5 |  |  |  | 0.36695 |  |
|  | | | | | | | | | | | | | |


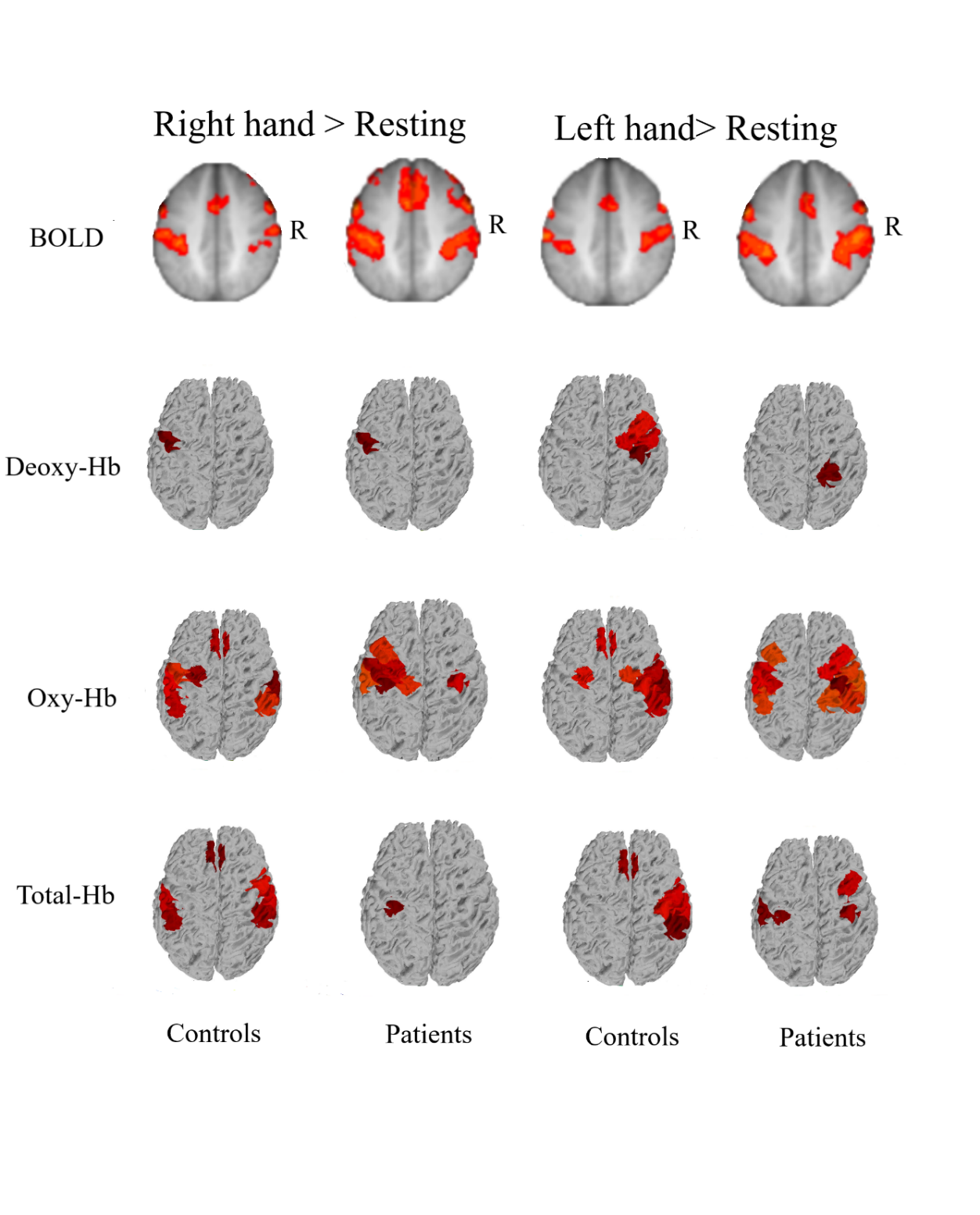


**Figure S7 Brain activation map during the finger-tapping task**: regions activated in both controls and patients relative to resting in fMRI (BOLD) p = 0.05, and fNIRS (Deoxy, Oxy and Total-Hb) p = 0.00217 (separately acquired) for right and left hand finger-tapping.
